# Commandant and Matron

**Published:** 1916-08-05

**Authors:** 


### THE MATRONS' AND SISTERS' DEPARTMENT.

### COMMANDANT AND MATRON.

The difficulties and complaints that have arisen in hospitals with respect to the positions and functions of commandant and matron are evidently due to neglect of certain fundamental principles of administration, which are probably unknown to the ladies, who in this matter are amateurs, who have taken up the work. The principles are quite few and quite plain, and if they had been observed no difficulties would have arisen.

I. *Power and Responsibility must go together.*—Every officer is responsible for those things which he or she has power to do, and is not responsible for things which he or she has no power to do. This is obvious, and everyone who understands it must agree to it. The difficulties that arise are due not to ignorance or disregard of the obvious rule, but to want of definition of the powers and duties of an officer. It is here that half the difficulties arise. If an officer does not know what she may do and what she may not, what she is responsible for and what she is not, what her powers are and what her duties are, there is sure to be chaos and confusion, conflict of authority, and work ill done. For this reason, the powers and duties of every official should be clearly explained. The explanation should not be deferred until the officer has been engaged and has entered on her duties: it should be made at the time of the engagement. Then there can be no mistake. It is clear from some of the letters that have appeared in the *Nursing Mirror* that some of the ladies who have taken up appointments of whose conditions they complain, entered on their engagement in ignorance of those conditions, and if they had known them would never have consented to take up the appointment. They were accustomed to some one or more institutions in which the officer called the matron had certain powers and certain duties, and they assumed, not very unnaturally, that in every institution the officer called by the same name has the same powers and the same duties. The assumption was natural, but it was erroneous. A matron in one institution has great powers and correspondingly great responsibilities. In another, though she is called by the same name, she occupies a very different position, with greatly restricted powers and correspondingly diminished responsibilities. If she finds herself in the one position when she expected to find herself in the other, the fault was hers in not having the position clearly explained to her on her engagement. Hence the second principle is that the duties of every officer should be clearly defined on engagement.

II. *Different Kinds of Work should be entrusted to different Officers.*—In a small institution, in which the officers are few, this cannot be done to the same extent as in a large institution in which the officers are many; but human capacity being limited as well as various, it is desirable that every officer should be put to that work which she can do best. The raising of funds and engaging the interest of outside people in the institution requires ability of a special kind, which the matron of a large hospital is not likely to possess, and which the kind of woman who takes the position of commandant is very likely to possess. She has probably been chosen for the very reason that she does possess the ability, and it must be remembered that an institution cannot be administered without funds, and that the financial is the aspect of the matter on which everything else depends. No money, no hospital. It is not unfair, therefore, that the officer chiefly responsible for the finances should be the titular head of the institution; but there is a third principle that is of equal importance.

III. It is that *a skilled person must not, in respect of the work in which she is skilled, be placed under the orders of an unskilled person.* To do this is to invite disaster. The skilled officer is indignant, and the unskilled officer is apt to be ridiculous; and when the subordinate officer can justifiably feel indignation and contempt for her superior, there is an end to harmony and also to efficiency in the institution. It is scarcely possible for discipline to be maintained in such circumstances, and it is quite impossible that in a hospital the patients should not suffer. It is obvious that it is the matron, not the commandant, who should accompany the doctor on his rounds, who should receive his instructions direct from himself, and who should in his absence control the nurses, the patients, the nursing, the diets, and everything to do with the nursing department.

If these principles are laid to heart and observed there need be no friction or disharmony between the commandant and the matron beyond what the general imperfection of human nature renders unavoidable; if they are not observed, there is sure to be confusion, cross-purposes, quarrels, lack of discipline, and injury to the interests of the patients.

